# Classical Examples of the Concept of the ASIA Syndrome

**DOI:** 10.3390/biom10101436

**Published:** 2020-10-12

**Authors:** Vânia Borba, Anna Malkova, Natalia Basantsova, Gilad Halpert, Laura Andreoli, Angela Tincani, Howard Amital, Yehuda Shoenfeld

**Affiliations:** 1 Zabludowicz Center for Autoimmune Diseases, Sheba Medical Center, Tel-Hashomer 5265601, Israel; vaniavborba@gmail.com (V.B.); Gilad.Halpert@sheba.health.gov.il (G.H.); Howard.Amital@sheba.health.gov.il (H.A.); 2 Laboratory of the Mosaic of Autoimmunity, Saint Petersburg State University, 5265601 Saint-Petersburg, Russia; anya.malkova.95@mail.ru (A.M.); fromrussiawithlove_nb@mail.ru (N.B.); 3 Sackler Faculty of Medicine, Tel-Aviv University, Tel-Aviv 6997801, Israel; 4 Department of Clinical and Experimental Sciences, University of Brescia, 25123 Brescia, Italy; laura.andreoli@unibs.it (L.A.); tincani@bresciareumatologia.it (A.T.); 5 Rheumatology and Clinical Immunology, ASST Spedali Civili, 25123 Brescia, Italy; 6 Ministry of Health of the Russian Federation, Sechenov First Moscow State Medical University, 119146 Moscow, Russia

**Keywords:** autoimmune diseases, ASIA syndrome, autoimmune/inflammatory syndrome induced by adjuvants, adjuvants, autoantibodies, silicone

## Abstract

Autoimmune/inflammatory syndrome induced by adjuvants (ASIA) was first introduced in 2011 by Shoenfeld et al. and encompasses a cluster of related immune mediated diseases, which develop among genetically prone individuals as a result of adjuvant agent exposure. Since the recognition of ASIA syndrome, more than 4400 documented cases have been reported so far, illustrated by heterogeneous clinical manifestations and severity. In this review, five enigmatic conditions, including sarcoidosis, Sjögren’s syndrome, undifferentiated connective tissue disease, silicone implant incompatibility syndrome (SIIS), and immune-related adverse events (irAEs), are defined as classical examples of ASIA. Certainly, these disorders have been described after an adjuvant stimulus (silicone implantation, drugs, infections, metals, vaccines, etc.) among genetically predisposed individuals (mainly the HLA-DRB1 and PTPN22 gene), which induce an hyperstimulation of the immune system resulting in the production of autoantibodies, eventually leading to the development of autoimmune diseases. Circulating autonomic autoantibodies in the sera of patients with silicone breast implants, as well as anatomopathological aspects of small fiber neuropathy in their skin biopsies have been recently described. To our knowledge, these novel insights serve as a common explanation to the non-specific clinical manifestations reported in patients with ASIA, leading to the redefinition of the ASIA syndrome diagnostic criteria.

## 1. Introduction

**Figure 1:**
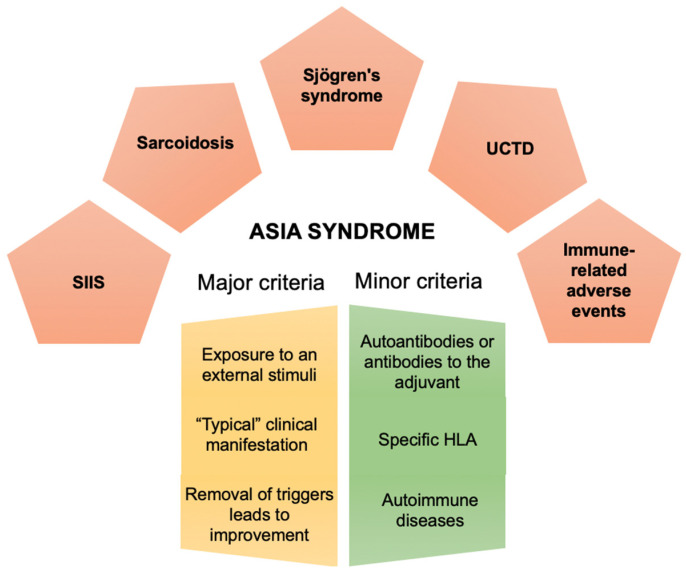
Diagnostic criteria for the ASIA syndrome and the five classical examples of this condition. (*SIIS*: silicone implant incompatibility syndrome; *UCDT*: undifferentiated connective tissue disease).

Autoimmune/Inflammatory syndrome induced by adjuvants (ASIA) was first introduced in 2011 by Shoenfeld et al. [1] and encompasses a cluster of immune mediated diseases, which are likely to develop among genetically predisposed individuals after the exposure to an adjuvant. These conditions share several clinical aspects with the possible appearance of autoantibodies, and trend to improve once the inciting agent is removed [2]. Clustering of autoimmune diseases (AID) in families is well recognized, supporting a common genetic background [3]. It is necessary that external environmental factors (infectious agents, dust, vaccines, etc.) or other adjuvant agents triggering immune activity (dust, silicone, aluminum salts, etc.) cooperate on this favorable genetically determined background, in order to promote the disease onset [4,5,6,7,8,9]. Notably, loci in the human leukocyte antigen (HLA), which have been shown to be associated with the development of AID have been suggested to be associated with the classical ASIA syndrome conditions [3,10]. Further, adjuvants influence both the innate and adaptive arms of the immune system via assorted mechanisms, encouraging the initiation and perpetuation of immune response by the activation of pattern recognition receptors. Nevertheless, enhanced immunogenicity might lead to reactogenicity in a process that does not always begin involving pathological stimulation [11]. Since the recognition of ASIA, more than 4400 documented cases have been reported so far, showing heterogeneous clinical manifestations and diverse severity [12]. Interestingly, several conditions, such as sarcoidosis, Sjögren’s syndrome, undifferentiated connective tissue disease, and silicone implant incompatibility syndrome, were seen to share pathogenetic aspects with ASIA syndrome (Figure 1).

Certainly, these disorders have been described after an adjuvant stimulus (vaccine compounds, silicone implantation, drugs, infections, metals, etc.) among genetically predisposed individuals (mainly the HLA-DRB1 and PTPN22 gene) [13,14], leading to an hyperstimulation of the immune system. As a result, the production of autoantibodies may occur, eventually driving the development of AID [15,16,17].

## 2. Classical Examples of the ASIA Syndrome

The establishment of the ASIA concept in 2011, has allowed to clarify the different pathways leading to the development of assorted autoimmune conditions considered so far to be “enigmatic”. Under the light of the recent discoveries, also supported by our own research results, disorders such as sarcoidosis, Sjögren syndrome (SS), undifferentiated connective tissue disease (UCTD), silicone implants incompatibility syndrome (mainly associated with silicone breast implants), and irAEs were able to be segregated as classical examples of the ASIA syndrome concept.

### 2.1. Sarcoidosis

Sarcoidosis is a systemic granulomatosis disorder of unknown etiology characterized by the formation of immune granulomas in various organs, mainly the lungs and the lymphatic system [18]. Studies have hypothesized that sarcoidosis might be the result of an exaggerated granulomatous reaction occurring after the exposure of a genetically prone individual to an unidentified antigen, that triggers a Th1-type cellular immune response leading to the formation of granulomas [19]. The hyperstimulation of the immune system is most probably prompted by an inorganic material, infection, environmental stimuli and/or autoantigens [20].

Several genome wide association studies have demonstrated that both HLA and non-HLA alleles are associated with the development of sarcoidosis and with disease phenotype [21] (Tables S1 and S2). There are at least eleven more relevant risk loci identified so far (chromosome 11q13.1, HLA-B, HLA-DPB1, ANXA11, IL23R, IL12B, BTNL2, NFKB1/MANBA, SH2B3/ATXN2, FAM177B, and RAB23). In this context, gene polymorphisms encoding for cytokines, chemokines and other molecules involved in inflammatory pathways, such as interleukin-1 (IL-1), tumor necrosis factor α (TNF-α), transforming growth factor β (TGF-β), and Toll-like receptor 4 (TLR-4), seem to be associated with disease susceptibility [19,21,22].

The exact trigger of sarcoidosis remains unknown. Immunologically, is an exaggerated immune response to an unidentified antigen. Isolation of *Mycobacteria* and *Propionibacteria* from tissue specimens collected from sarcoidosis patients suggests that these pathogens may represent strong candidates for infection-mediated sarcoidosis, since host response promotes the aggregation and persistence of the non-degradable antigens, collecting a nidus for granuloma formation [23]. Indeed, antibodies to mycobacterial proteins p36, heat shock proteins 65 and 70 were found among these patients. Likewise, hepatitis C patients treated with interferon-α therapy, which increases interferon-γ (IFN-γ) and IL-2 expression, have been reported to develop sarcoidosis. In addition to infections, other environmental factors, such as insecticides, dust, pollens, inorganic particulates, etc., have also been linked to disease onset. More recently, a role for silicone has been suggested [24]. A large study performed by Watad et al. comparing 24,651 patients with silicone breast implants and 98,604 controls, showed an increased risk of autoimmune diseases related to silicone (OR 1.21, 95% CI 1.17–1.26). The strongest associations were observed with sarcoidosis, SS, and systemic sclerosis (SSc) [25]. Interestingly, giant cell granulomas containing silicone particles were found at implantation *loci*, and the described changes regress in 60–80% of cases after silicone explant [19]. More recently, vimentin (a peptide which is involved in intercellular interactions and functioning of the immune system, involved in the pathogenesis of inflammation and many autoimmune diseases) [26], has been detected inside the giant multinucleated cells of sarcoid granulomas, as well as associated with specific T-cells and antibodies to vimentin in representatives of the HLA-DRB1*0301 genotype [27,28]. A disturbance of cellular and humoral immune response has been described in sarcoidosis, since the triggering factor for inflammation is the contact of the antigen with antigen-presenting cells, leading to activation of T and B lymphocytes which migrate to the inflammatory site [29,30]. The growth of granulomas, containing macrophages and their derivatives, T cells, giant cells and epithelioid cells, establishes the primary abnormality in most cases of sarcoidosis. The resolution or maintenance of granuloma is determined by the proportion of Th1 and Th2 cells, respectively. Likewise, macrophages begin to differentiate into M2 type, which have anti-inflammatory properties and contribute to the chronicity of the process and fibrosis. Polyclonal hypergammaglobulinemia has been observed among these patients, although an association with the disease course has not been established [31]. In recent years, an imbalance of T helper lymphocytes and their subpopulations (Th1/Th17 or Th17.1 cells) [32], capable of producing IL-17 and IFN-γ, have been linked to the pathogenesis [33,34,35]. Increased levels of autoantibodies, including antinuclear (ANA), anti-cyclic citrullinated peptide antibodies (anti-CCP), and anti-ds-DNA in the serum of sarcoidosis patients, have been reported in several studies [36]. Further, the clinical improvement observed with anti-B-cell drugs, suggest that B cells might also play an important role [37,38]. Interestingly, up to 86% of patients present with typical small fiber neuropathy (SFN) symptoms, considered to be of systemic cytokine-mediated nature rather than of granulomas occurrence [39]. Nevertheless, immunomodulatory therapies involving TNF-α inhibition have shown beneficial results, improving the clinical manifestations of SFN in sarcoidosis patients [40]. In this manner, it is believed that sarcoidosis might begin as an inflammatory disease, evolving to an activation of the adaptive immunity under the influence of several triggering factors, which are conducive to a constellation of immunological reactions characteristic of autoimmune diseases [41].

### 2.2. Silicone Implant Incompatibility Syndrome

Women with silicone-related complaints due to SBIs have been included in the classical models of ASIA syndrome. The silicone present in the breast implants represent an external non-self, chronic stimulus that may lead to hyperstimulation of the immune system in genetically predisposed individuals, appearance of non-specific subjective clinical manifestations, and autoantibody production, which might precede the development of autoimmune diseases, and most rarely lymphoma.

Silicone injections and the subsequent use of SBIs for breast reconstruction and breast augmentation have been reported since 1960’s [42,43,44]. The safety of silicone breast implants has stirred an intense debate, concerning their potential for induction of autoimmunity and lymphoma [15,45,46,47,48,49,50,51,52,53]. The expression of HLA-DRB1 and HLA-DQ alleles in patients with SBIs can be related with the development of autoimmune symptoms [54,55,56,57]. Several plausible mechanisms have been proposed to explain the link between SBIs and autoimmune phenomena, as it has been shown in animal model studies. For example, injection of silicone-gel in NZB mice has led to the induction of proteinuria and autoimmune hemolytic anemia, whereas implantation of silicone-gel or silicone oil in MRL lpr/lpr mice has led to the increase of anti-ds-DNA antibodies [58,59]. We have previously shown that silicone can trigger UCTD, SSc and fibromyalgia [47,60,61]. Moreover, in a large population-based study, we have recently demonstrated an association between SBIs and the presence of autoimmune/rheumatic disorders such as SS, SSc, and sarcoidosis [25]. Furthermore, we have reported an increased production of a broad range of autoantibodies in asymptomatic and symptomatic women with SBIs [62]. These autoantibodies may predict and precede the development of autoimmune disease in these women. The complex link between SBIs and autoimmunity can be illustrated by the concept of ASIA syndrome [1,48,49,63]. Recently, our group has diagnosed almost 100 women with SBIs, suffering from classical ASIA-related, non-specific, diverse clinical manifestation as: chronic fatigue, sleep disturbance, widespread pain, memory loss, dry mouth and eye, cognitive impairment, tachycardia, hearing abnormalities, allergic reaction, depression, hair loss, irritable bladder and bowel syndrome, palpitations etc. Such a wide array of manifestations was frequently misdiagnosed by several physicians who examined those patients, basing their conclusions in normal diagnostic tests (routine serologies, electromyography, etc.).

Regarding the proposed criteria for the diagnosis of ASIA syndrome, we previously suggested the appearance of non-specific autoantibodies (ANA, anti-thyroglobulin antibodies etc.) and/or antibodies directed against the suspected adjuvant [1]. In accordance with a recent study conducted at our center, we found two objective phenomena in women with silicone breast implants:

1. Circulating autoantibodies against G protein-coupled receptors of the autonomic nervous systems such as adrenergic and muscarinic acetylcholine receptors have been found in the sera of these women, which we believe, may explain, at least in part, some of the enigmatic, subjective and undefined clinical manifestations reported by these women (personal communication). It is also worth mentioning that some studies reported that removal of the silicone breast implants led to significant improvements in clinical manifestation [64,65].
2. Five women with SBIs have been diagnosed with SFN after evaluation of their skin biopsies. These two new objective findings (autoantibodies against the autonomic nervous system and SFN) have been found both in other ASIA-related entities (sarcoidosis, SS etc.) and other suspected autoimmune dysautonomic-related disorders. Therefore, it should be regarded as a new objective criterion of ASIA syndrome [17].

Our recent findings regarding the appearance of circulating autonomic autoantibodies and SFN might serve as a common explanation to the non-specific clinical manifestations reported by women with SBIs and other ASIA-related entities. They could also serve to re-define the ASIA diagnostic criteria.

### 2.3. Sjögren’s Syndrome

SS is a chronic systemic autoimmune inflammatory condition primarily involving the exocrine glands in which both genetic and environmental factors play a pathogenic role. Infections represent the most prominent trigger of disease [66] leading to a dysregulated immune response largely driven by an overexpression of type I interferons, B cell proliferation, aberrant cytokine production, and tissue infiltration. Recent evidence suggests that several agents may act as adjuvants in determining such abnormal immune response possibly contributing to the development of SS [67,68]. Vaccinations, which should follow a recommended schedule in patients with autoimmune diseases including patients with SS, should preferably be administered during quiescent phases of the diseases due to the possibility to trigger a disease flare [69,70] and for same reason live attenuated vaccines should be avoided. Some studies suggested that SS onset can be associated with specific vaccines, still a temporal rather than a causal association should always be considered [71,72,73]. Nonetheless, it was shown that, in patients with primary SS, the A/California/7/2009/H1N1-like virus vaccination lead to a significant increase in the mean levels of anti-SSA/Ro and anti-SSB/La antibodies after 1-year of follow-up [74]. Alum, an aluminium-based adjuvant, was able to induce a Sjögren’s syndrome-like disease in an experimental New Zealand Mixed (NZM) 2758 strain of mouse in which ANA positivity, chronic salivary gland dysfunction and lymphocytic infiltrates within the salivary glands was observed. Other possible triggers of SS development could be silica and silicone that may stimulate a polyclonal B cell activation with local production of cytokines through chronic inflammation. There are few reports suggesting that silica exposure can precede the onset of SS especially in certain chronically exposed professional groups, such as dental technicians [75,76] and coalminers [77,78,79]. The association between silicone breast implants and autoimmune diseases still remains inconclusive considering data from systematic reviews and meta-analyses [80,81,82]. A more recent post-approval long-term outcome study on an extremely large cohort suggests that there are higher rates of SS and other autoimmune disorders in patients undergoing silicone breast implants [83]. This is consistent with case reports dating back 1984 and with the several case series reported over time [84]. Data from a cross-sectional real-world analysis matching woman who underwent silicone breast implants and autoimmune disorders, highlighted an association between this procedure and SS [25]. In another study the authors found that patients complained more SS-related symptoms at one-year follow-up after breast reconstruction even if there was no correlation with autoantibodies or imaging (magnetic resonance) changes of the implants [85]. Indeed, in an autoimmune setting, jumping into a patient who has recently undergone breast implant surgery can sporadically occur. The most relevant issue is, again, that the association could be temporal rather than causal. Cohen Tervaert et al. [86,87] followed a number of patients who were identified with silicone implant incompatibility syndrome as part of ASIA, who developed, among the other full-blown autoimmune diseases, SS. Interestingly, most patients ameliorated after replacing silicone-filled breast implants by saline–cellulose-mixed implants [88], possibly suggesting a causal association. Alijotas-Reig et al. observed that 15 out of 185 cases (8%) of patients suffering from inflammatory/autoimmune disorders related to bioimplant injections had systemic or distant and multiple complaints that could be categorized as ASIA. SS was diagnosed in two patients representing a rather infrequent yet possible manifestation following a chronic stimulation from biomaterials used as fillers [89], an evidence reinforced by a further 3 cases published later on [90]. Thus, the epidemiology and pathomechanisms possibly linking some agents acting as adjuvants such as vaccines, silica, silicone implants and other biomaterials with SS are yet to be clarified. This will allow to prevent the onset of SS in susceptible predisposed individuals and to adopt personalized therapeutic strategies.

### 2.4. Undifferentiated Connective Tissue Disease

Several conditions in the field of autoimmunity are characterized by non-specific signs and symptoms that cannot be classified into a definite nosological entity according to international criteria. An increasing number of patients have been referred to rheumatology consultation for chronic fatigue, myalgia, muscle weakness, arthralgia/arthritis, and interstitial lung disease [91,92]. The term ‘undifferentiated’ used to describe all these conditions not only reflects an undefined clinical picture but also a poor knowledge of the underlying etiopathogenic mechanisms. UCTD is a term that encompasses a broad spectrum of conditions characterized by signs, symptoms and laboratory features that are suggestive of systemic autoimmune diseases (SADs) [93]. Such a kaleidoscope of clinical presentations poses the question whether the UCTD can be considered as a distinct entity or may be early forms of definite SAD, which is the reason why the classification criteria for UCTD are still a work in progress [94].

The induction and perpetuation of autoimmunity is a complex process that requires the interaction between the genetic background and the environment. Environmental factors are gaining increasing attention in the pathogenesis of UCTD. Similar to ASIA [95,96], UCTD is an autoimmune condition characterized by non-specific signs and symptoms, alluding to the idea that the exposure to adjuvants can be a trigger of UCTD. To investigate the possible environmental triggers of UCTD, a case–control study on the exposure to different adjuvants in 92 patients with UCTD and in 92 age and sex-matched controls was performed in Italy [61]. Exposure to several adjuvants prior to UCTD onset (during the 10 years before diagnosis) was found to be significantly more frequent than healthy controls, suggesting that nearly half of UCTD patients in our cohort might fall within the spectrum of ASIA. Interestingly, patients exposed to major adjuvants (vaccines containing adjuvants or silicone implants) displayed the typical features of ASIA, particularly fibromyalgia symptoms (Table 1). The association between vaccinations and autoimmune phenomena has been described as either simple appearance of autoantibodies or as a full-blown autoimmune disease [97,98]. The onset of UCTD has been described following hepatitis B vaccination [99,100]. The relationship between vaccinations and systemic autoimmune diseases has been reported also for SS [67] and the antiphospholipid syndrome [101]. The magnitude of autoimmune post-vaccination phenomena is minimal against the number of vaccinated individuals who did not develop any autoimmune complication. This can be explained by the presence of a predisposing genetic background, being HLA-DRB1 a prototypical example [102]. The International ASIA Syndrome Registry has been collecting hundreds of cases. Interestingly, the most frequent autoimmune disease related to ASIA syndrome was UCTD [60] and an association between polygenic autoimmune diseases and HBV/influenza vaccinations was found [16].

The kaleidoscopic spectrum of UCTD can include patients who fulfil the definition of ASIA syndrome. Therefore, clinicians should investigate their patients for environmental exposures and carefully evaluate whether any trigger can be removed or attenuated in order to down-modulate the altered immune response.

### 2.5. Immune-Related Adverse Events

The irAEs are autoimmune complications of check-point inhibitors (CPI) therapy used in cancer treatment. The main difference of IrAEs in comparison with AID is a lack of the chronicity [103]. It is one of the ASIA classical example where the external stimuli are known, and its pathogenesis is well described. In this case “adjuvants” are monoclonal antibodies, that inhibit a receptor associated with cytotoxic T lymphocytes (CTLA-4), a programmed cell death receptor-1 (PD-1), and its main ligand PD-L1. The blockade of control points CTLA-4 and PD-1 reduces the prevention against autorecognition by lymphocytes and contributes to activation of CD-8+ and CD4+ T cells against cancer cells. This overstimulation of immune system breaks the auto-tolerance and leads to autoimmune reactions [104]. According to clinical trials irAES develop up to 90% of patients treated with an anti-CTLA-4 antibody and 70% of patients treated with an PD-1/PD-L1 antibody [105,106]. The median onset is 3–6 months after the start of treatment. However, late adverse events, which occur after a year or more, are also documented [107]. In mild cases the symptoms might disappear by its own or after termination of CPI exposure, but severe irAEs needs to be managed with immune-modulatory medications, such as steroids, biological therapy, or cytostatic drugs [108,109,110]. In addition, the characteristic ASIA symptoms can occur in patients who have developed musculoskeletal toxicities, which are found in 2–12% of cases and can manifest as inflammatory arthritis, myalgia, myositis, and syndromes similar to polymyalgia [111]. Fever is a common complication of immunotherapy. In patients with non-small cell lung cancer, fever was associated with a low level of progression free survival [112]. Chronic fatigue, not-restful sleep, sleep disturbances, cognitive alterations, memory loss occur in up to 42% of cases and may be associated with immune activation in the central nervous system [113]. It should not be forgotten that the described symptoms can be a manifestation of endocrine abnormalities [114].

Further, during the therapy autoantibodies appear in many patients, and often there is no association with particular organ affection. In some cases, the appearance of autoantibodies is associated with a good prognosis [115,116]. Some researchers managed to find organ-specific autoantibodies, but this is more likely due to the development of a chronic disease. Researchers of the Salahaldin A. Tahira group determined the organ-specific antibodies in the development of hypophysitis, namely anti-GNAL and anti-ITM2B, and pneumonitis, anti-CD74 [117], while antibodies to thyroperoxidase are used for autoimmune thyroiditis diagnosis [118]. Despite the contradictory results, some authors suggest using ANA, anti-smooth muscle antibody, for the diagnosis of liver damage. For lung disorders, they suggest ANA, rheumatoid factor (RF) and extractable nuclear antigen (ENA), and ANA, ENA, and anti-CCP to diagnose polyarthritis [105].

Another key aspect is the association with specific HLA (i.e., HLA DRB1, HLA DQB1). According to Omar et al., a single locus of predisposition to the development of irAEs was not found, but the researchers were able to find a significant association between HLADRB1*11:01 and pruritus, HLA-DQB1*03:01 and colitis in patients with metastatic non-small cell lung cancer and metastatic melanoma, who were treated with anti-PD-1, anti-CTLA4 or both drugs combined [119]. Currently, researchers are trying to find a correlation between the known genotypes associated with autoimmune diseases and the possibility of developing certain autoimmune complications in patients taking control point inhibitors [120].

Due to the fact that patients with preexisting autoimmune diseases had a higher frequency of severe irAEs and new disease symptoms, for some time there were restrictions on the inclusion of these patients in treatment groups [121]. However, recent retrospective data have demonstrated the safety and efficacy of ICIs in patients with pre-existing AID [122]. The presence of AID in the anamnesis requires more careful monitoring of the patient’s condition, and the management with specialists in autoimmune pathologies. At the moment, the difficulty is defined by a preclinical autoimmune condition in patients with asymptomatic autoantibodies. Patients with preexisting RF, ANA, antithyroglobulin, or antithyroid peroxidase have been noted to be significantly more susceptible to irAE development [123]. Nevertheless, it is difficult to say which of the described complications are reversible and which then became chronic AID [110]. There are studies showing that the development of symptoms of an inflammatory SADs, such as inflammatory arthritis, myositis, SS, or vasculitis, occurs in approximately 3.5–6.6% of patients treated with CPI [124]. Patients with rheumatoid arthritis can have erosive changes on radiographs and may have positive serology for RF and anti-CCP antibodies. A ‘seronegative’ group present with synovitis of medium and large joints [125]. Vasculitides such as giant cell arteritis and polymyalgia rheumatica, SS and systemic lupus erythematosus are less common (<1%) [126]. The data about developed autoimmune endocrine diseases have also been obtained. According to studies thyroiditis or Grave’s disease is a rare complication of CPI therapy [114]. Diabetes mellitus can be found in 3% of patients. The pathophysiology has not yet been fully elucidated but there is likely involvement of CD8 + T cell response to T1DM antigen and type 1 diabetes-specific autoantibodies (GAD65) [127]. See also Table S1 [127,128,129,130,131,132,133,134].

## 3. Redefining ASIA Syndrome Concept

ASIA syndrome is a recently identified condition (2011), which associates the occurrence of autoimmune/autoinflammatory diseases following an exposure to adjuvants. Nevertheless, given its rarity, an international registry has been created in order to systematize and improve our knowledge about this heterogeneous entity. Recently, a large cohort analysis of 500 patients in the ASIA registry performed by our group (2017, 2019), provided crucial new insights about potential adjuvants, clinical manifestations, antibody profile and associated autoimmune diseases [16,60]. Furthermore, we recently found the appearance of circulating autonomic autoantibodies (autoantibodies against adrenergic receptors, acetylcholine receptors, muscarinic receptors, G-protein coupled receptors and angiotensin II receptor type 1) and anatomopathological aspects of small fiber neuropathy (SFN) in skin biopsies of patients with silicone breast implants (SBIs) [16,17].

**Figure 2:**
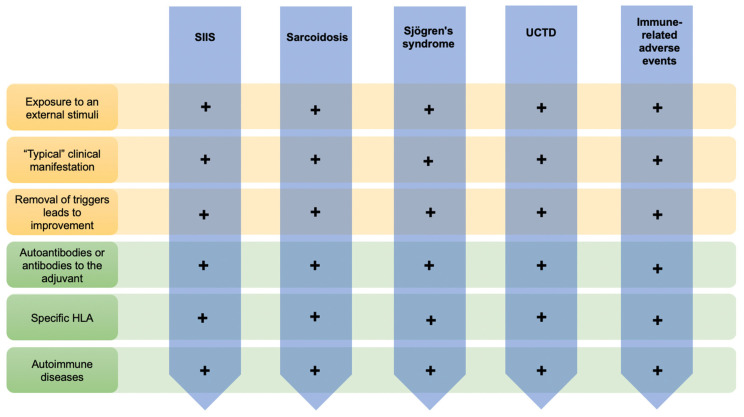
The classical examples of ASIA syndrome—fulfill the suggested criteria for ‘ASIA’ syndrome. (*SIIS*: silicone implants incompatibility syndrome; *UCDT*: undifferentiated connective tissue disease).

To date, our acumen regarding ASIA has allowed not only to reinforce the proposed diagnostic criteria (Table S3), but also to provide a common explanation to the non-specific clinical manifestations reported in these patients and decode the previous mentioned maladies as classical examples of the ASIA concept (Figure 2).

## 4. Conclusions

ASIA syndrome encompasses various autoimmune conditions that flourish under the influence of triggering factors among predisposed individuals, which provoke immunological reactions characteristic of autoimmune diseases. Conditions for which etiology is so far beyond comprehension, such as sarcoidosis, Sjögren’s syndrome, UCTD, silicone implant incompatibility syndrome, and immune adverse related events, represent classical examples of the ASIA syndrome. The described major (clinical) and minor (immunogenetic) diagnostic criteria enable us to assume the autoimmune nature of inflammation seen in these diseases. The harmful role of adjuvants has already been recognized in the scientific community, and although vaccines contain adjuvants, it is extremely important to highlight that general benefits of vaccination far outweigh the risk of immune-related side effects. In this manner, efforts should be made in order to understand, clarify, and raise the awareness of clinicians regarding the ASIA concept, for a better discernment between the adjuvant-induced pathologies and their prevention among genetically predisposed individuals. They could test for specific genetic markers (largely unknown) for every vaccination. Nevertheless, some open questions remain and should be addressed in future studies, such as “Why is there autonomic dysfunction and neuropathy in women with SBIs as apparent in the production of autoantibodies against specific receptors of the autonomic nervous system and SFN?”.

## Figures and Tables

**Table 1:** Features linking undifferentiated connective tissue disease and ASIA syndrome in a case–control study on environmental exposures [61]. (*UCDT*: undifferentiated connective tissue disease).

Shared Features between UCTD and ASIA	Main Findings
(A) UCTD patients had more environmental exposures to adjuvants as compared to controls.	UCTD were significantly more exposed to: (1) tetanus vaccination; (2) HBV vaccination; (3) metal implants; (4) proximity to metal factories and foundries (home located less than 1 km). Cigarette smoking and allergies were more frequent in UCTD.
(B) Half of UCTD exposed to major ASIA triggers.	Fifty-seven percent of patients with UCTD had been exposed to either vaccines containing adjuvants or silicone implants.
(C) UCTD exposed to major ASIA triggers displayed typical features of ASIA.	As compared with non-exposed UCTD patients, those exposed to major ASIA triggers displayed more frequently general weakness, chronic fatigue, irritable bowel syndrome.
(D) UCTD exposed to major ASIA triggers had familiarity for autoimmunity.	As compared with non-exposed UCTD patients, those exposed to major ASIA triggers had more frequently first-degree relatives with autoimmune diseases (56% vs. 33%).

## Supplementary Materials

The following are available online at https://www.mdpi.com/2218-273X/10/10/1436/s1, Table S1: HLA genotypes influencing sarcoidosis clinical presentation and prognosis; Table S2: Summary of major animal studies of probiotic interventions, their mechanism of action and their outcomes related to ASD; Table S3: Summary of major clinical studies of probiotic interventions, their mechanism of action and their outcomes related to ASD.

## Abbreviations

AID	Autoimmune diseases
ANA	Antinuclear antibody
Anti-CCP	Anti-cyclic citrullinated peptide
ASIA	Autoimmune/Inflammatory syndrome induced by adjuvants
ASMA	Anti-smooth muscle antibody
CI	Confidence interval
CPI	Check-point inhibitors
CTLA	Cytotoxic T lymphocyte antigen
ENA	Extractable nuclear antigens
HBV	Hepatitis B virus
HLA	Human leukocyte antigen
ICIs	Immune checkpoint inhibitors
IFN-γ	Interferon gamma
IL	Interleukin
irAEs	Immune-related adverse events
NZB	New Zealand black
NZM	New Zealand mixed
OR	Odds ratio
PD-1	Programmed cell death receptor-1
PFS	Progression free survival
RF	Rheumatoid factor
SADs	Systemic autoimmune diseases
SBIs	Silicone breast implants
SFN	Small fiber neuropathy
SIIS	Silicone implant incompatibility syndrome
SS	Sjogren’s syndrome
SSc	Systemic sclerosis
TGF-β	Transforming growth factor β
Th	T helper lymphocytes
TLR-4	Toll-like receptor 4
TNF-α	Tumor necrosis factor α
UCTD	Undifferentiated connective tissue disease
