# Supplementary material for: Classical Examples of the Concept of the ASIA Syndrome
Source: Biomolecules. 2020 Oct 12;10(10):1436. doi: 10.3390/biom10101436 (PMC7600067; doi:10.3390/biom10101436)
Supplement: Supplementary file 1 [file biomolecules-10-01436-s001.pdf]

Supplementary materials

Review

Classical Examples of the Concept of the ASIA Syndrome

Vânia Borba [1], Anna Malkova [2], Natalia Basantsova [2], Gilad Halpert [1,3], Laura Andreoli [4,5], Angela Tincani [4,5,6], Howard Amital [1,3], and Yehuda Shoenfeld [1,2,3,*]

[1] Zabludowicz Center for Autoimmune Diseases, Sheba Medical Center, 5265601Tel-Hashomer, Israel

[2] Laboratory of the Mosaic of Autoimmunity, Saint Petersburg State University, Saint-Petersburg, Russian Federation
[3] Sackler Faculty of Medicine, Tel-Aviv University, Tel-Aviv, Israel
[4] Department of Clinical and Experimental Sciences, University of Brescia, Brescia, Italy
[5] Rheumatology and Clinical Immunology, ASST Spedali Civili, Brescia, Italy
[6] Sechenov First Moscow State Medical University of the Ministry of Health of the Russian Federation

* Correspondence: shoenfel@post.tau.ac.il

Received: 17 July 2020; Accepted: 25 September 2020; Published: 12 October 2020

Table S1. HLA genotypes influencing sarcoidosis clinical presentation and prognosis.

HLA Genotype	Disease Characteristics	Ref.
DRB1*01/04, DRB1*03:01, B08	Acute course, Löfgren´s syndrome	[22,128,129]
DRB1*01, DRB1*03	Löfgren´s syndrome, favorable prognosis	[131,132]
DRB1*03, DQB1*02:01	Acute course, favorable prognosis	[133,134]
DRB1*15, DQB1*06:02	Chronic course	[133,134]
DRB1*07, DRB1*14, DRB1*15	Chronic course, poor prognosis	[131]
DRB1*04/15, DRB1*12/14, DRB1*14:01	Chronic course, extra-pulmonary lesions	[130]

Table S2. Summary of major animal studies of probiotic interventions, their mechanism of action and their outcomes related to ASD.

Shared Features Between UCTD and ASIA	Main Findings
(A) UCTD patients had more environmental exposures to adjuvants as compared to controls.	UCTD were significantly more exposed to: (1) tetanus vaccination; (2) HBV vaccination; (3) metal implants; (4) proximity to metal factories and foundries (home located less than 1 km). Cigarette smoking and allergies were more frequent in UCTD.
(B) Half of UCTD exposed to major ASIA triggers.	Fifty-seven percent of patients with UCTD had been exposed to either vaccines containing adjuvants or silicone implants.
(C) UCTD exposed to major ASIA triggers displayed typical features of ASIA.	As compared with non-exposed UCTD patients, those exposed to major ASIA triggers displayed more frequently general weakness, chronic fatigue, irritable bowel syndrome.
(D) UCTD exposed to major ASIA triggers had familiarity for autoimmunity.	As compared with non-exposed UCTD patients, those exposed to major ASIA triggers had more frequently first-degree relatives with autoimmune diseases (56% vs. 33%).

Table S3. Summary of major clinical studies of probiotic interventions, their mechanism of action and their outcomes related to ASD.

**Major criteria**
Exposure to an external stimulus (infection, vaccine, silicone, adjuvant) prior to clinical manifestations
Appearance of "typical" clinical manifestations
Myalgia, myositis, or muscle weakness
Arthralgia and/or arthritis
Chronic fatigue, un-refreshing sleep, or sleep disturbances
Neurological manifestations (especially associated with demyelination)
Cognitive impairment, memory loss
Pyrexia, dry mouth
Removal of inciting agent induces improvement
Typical biopsy of involved organs
**Minor criteria**
Appearance of autoantibodies or antibodies directed at the suspected adjuvant
Specific HLA (i.e., HLA DRB1, HLA DQB1)
Evolvement of an autoimmune disease (i.e., multiple sclerosis, systemic sclerosis)
Other clinical manifestations (i.e., irritable bowel syn.)
